# Fatigue behavior of removable partial denture cast and laser‐sintered cobalt‐chromium (CoCr) and polyetheretherketone (PEEK) clasp materials

**DOI:** 10.1002/cre2.645

**Published:** 2022-08-08

**Authors:** Jenny Zheng, John M. Aarts, Sunyoung Ma, John N. Waddell, Joanne J. E. Choi

**Affiliations:** ^1^ Faculty of Dentistry, Sir John Walsh Research Institute University of Otago Dunedin New Zealand

**Keywords:** CAD/CAM, Cast CoCr, denture clasps, fatigue, laser‐sintered CoCr, PEEK, removable partial denture

## Abstract

**Objective:**

To investigate the fatigue behavior of cast and laser‐sintered cobalt‐chromium (CoCr) and polyetheretherketone (PEEK) material for a removable partial denture (RPD) clasps.

**Methods:**

Dumbbell‐shaped specimens were digitally designed with the center part of the dumbbell being a half‐round shape at the cross‐sectional dimension of 1.25 mm to simulate a typical clasp design and dimensions. A fatigue machine with an offset axis rotation system simulated a typical undercut depth of 0.25, 0.50, and 0.75 mm. Each group was subjected to 30,000 fatigue cycles (simulating 21 years) or till specimen failure. Before testing, the stress value at each undercut depth for each specimen was established in a universal testing machine and SN curves were plotted for each group. Data were statistically analyzed using Kruskal–Wallis and post hoc tests. The fractured surfaces were analyzed using SEM.

**Results:**

The average fatigue cycles with 0.25, 0.50, and 0.75 mm undercuts were 27,155 ± 6277, 9298 ± 4033, 5642 ± 8785 for cast CoCr and 26,765 ± 6150, 11,318 ± 7931, 2861 ± 4803, for laser‐sintered CoCr, respectively. Apart from three specimens, the PEEK groups did not fail during the simulation period. Clasps with 0.25 mm deflection showed significantly higher fatigue resistance than other groups (*p* < .001). There was no significant difference in fatigue behavior between the cast and laser‐sintered CoCr. Microporosities at the fractured site along with irregular crack propagation were observed for cast and laser‐sintered CoCr specimens. Fatigue‐induced broken polymer crosslinking chains were observed in PEEK specimens.

**Conclusion:**

PEEK material exhibited the highest fatigue resistance and significantly lower deflection resistance. Cast and laser‐sintered CoCr showed similar fatigue resistance and behavior.

## 1 INTRODUCTION

Since the 1950s, the introduction of cast clasp‐retained removable partial dentures (RPDs) has been well established as a treatment option to restore function and aesthetics for patients (Pospiech et al., 2012).

Traditional methods of casting removable partial denture frameworks are well established; however, there are challenges with manual castings that can result in potential inaccuracy, which can impact the properties of the material (Pospiech et al., 2012). An advantage that computer‐aided design and additive manufacturing protocols can offer is that to a large degree, they overcome the disadvantages that casting alloys tend to incur. Laser sintering technology allows the fabrication of framework with high precision, competitive cost, and arguably more reliable results. The other disadvantage of traditional chrome castings is the poor aesthetics due to their metallic color and as a result, resin‐based polymer materials have come onto the market in an attempt to provide a more aesthetic material. However, it has been suggested that resin‐based polymer material's inherent mechanical properties mean that to achieve sufficient retention, a clasping unit needs to be bulkier than a metal clasping unit (Akl & Stendahl, 2022; Pospiech et al., 2012; Vermeulen et al., 1996; Zhao & Wang, 2014). From a biomechanical point of view, support and retention are key considerations to achieving a successful outcome. Direct retainers consist of a clasp assembly that directly interacts with the abutment teeth to provide retention, resistance, and stability (Zhao & Wang, 2014). However, a retentive clasp undergoes repeated stress caused by dynamic movement in the mouth including insertion and removal of the RPD. The failure of the retentive clasp has been reported as a complication (Vermeulen et al., 1996). Past studies stated that 15% of RPD repairs were caused by a fracture of the metal framework, while 19% of cast cobalt‐chromium (CoCr) RPD clasps fractured after an average of 4.5 years (Spiekermann, 1975). Cast CoCr has historically been a widely used method for fabricating RPD metal frameworks; however, new technologies and materials have been recently introduced for the manufacture of RPDs, such as laser‐sintered CoCr and polyetheretherketone (PEEK) (El‐Baz et al., 2020; Kajima et al., 2016; Peng et al., 2019; Schweiger et al., 2020; Tannous et al., 2012). The laser‐sintering utilizes a laser to fuse metal alloy powders layer by layer in a 3D additive technique, (Khurshid et al., 2019) while the PEEK material is manufactured via a subtractive milling technique (Tamimi & Hirayama, 2019).

A systematic review by Zheng et al. (2021) highlighted the lack of research available on the fatigue behaviors of the RPD clasps made with PEEK, cast, and laser‐sintered CoCr alloys. There was also a lack of standardized testing methods in the previous studies used to evaluate the fatigue life of the RPD clasps, which were mostly vertical insertion/removal methods and constant deflection tests. Studies that used vertical insertion/removal methods normally involved a universal testing machine, which directed the clasp assembly to engage and disengage with the tooth in a continuous cyclic motion. A limitation of this method was that the stress applied to the various parts of the tapered clasps was not calculated/established before failure, nor was the effect of friction established (Cheng et al., 2010; El‐Baz et al., 2020; Peng et al., 2019; Rodrigues et al., 2002; Schweiger et al., 2020; Tannous et al., 2012; Tokue et al., 2013). The constant deflection testing methods used tapered specimens, (Cheng et al., 2010; El‐Baz et al., 2020; Iwama & Preston, 1997; Kajima et al., 2016; Mahmoud et al., 2005; Mahmoud, 2007; Peng et al., 2019; Rodrigues et al., 2002; Schweiger et al., 2020; Tannous et al., 2012; Tokue et al., 2013; Vallittu & Kokkonen, 1995) resulting in a nonuniformed cross‐section area making analysis of failure in terms of stress at the point of fracture difficult (Murakami et al., 2021). The lack of standardization and the unidirectional force was a limitation when investigating the fatigue behavior of RPD clasps.

Therefore, the purpose of this study was to investigate the SN (the number of cycles to failure) fatigue behavior of cast CoCr, laser‐sintered CoCr, and PEEK for RPD clasps in terms of typical undercut deflections using a rotating fatigue system. The two null hypotheses (H_0_) were: first, that there would be no difference in fatigue resistance between the clasp materials and second, that there would be no difference in fatigue resistance between the amount of deflection of the different clasp materials.

## 2 MATERIALS AND METHOD

### 2.1 Materials and specimen preparation

**Figure 1:**
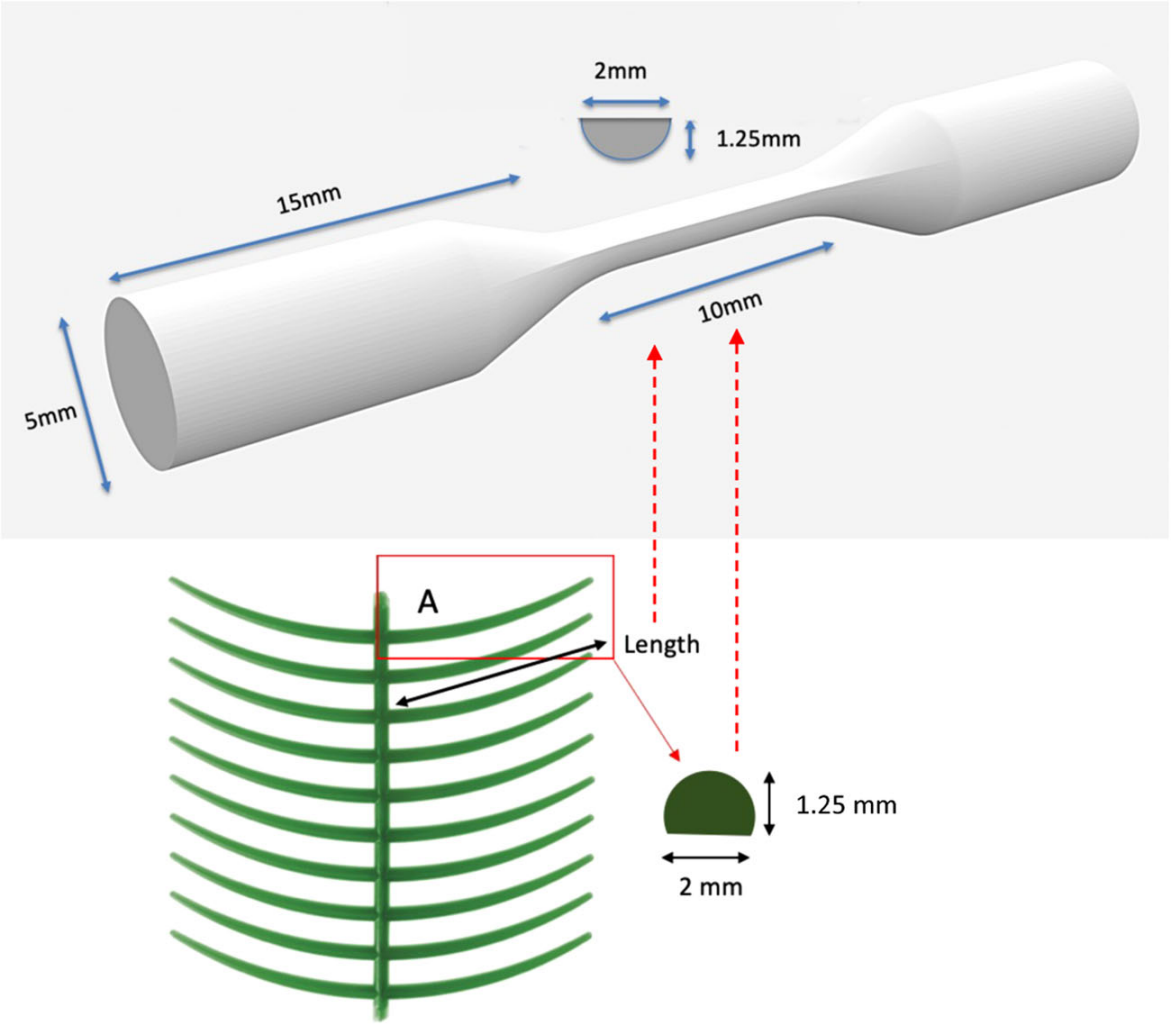
Shape and dimensions of dumbbell test specimens. The diagram shows the dimension of a typical preformed wax pattern, which will be replicated in test specimens.

A dumbbell specimen was created (according to the dimension outlined in ISO 1143:2021; Figure 1) via a computer‐aided design system to produce a standard triangulation language (STL) format file. This pattern was used to mill the laser‐sintered and milled PEEK specimens and 3D print resin burnout patterns for the cast CoCr technique (Figure 1). The materials used are listed in Table 1. The digital design file (STL format) of specimens for testing was generated in a dumbbell shape. The end regions were made substantially larger in cross‐section to withstand the forces of gripping and to avoid failure occurring within the grip section. The transition from the ends to the central portion is gradual, with well‐rounded corners to avoid points of stress concentration, which could lead to failure at these locations. The central region of the specimen was uniform, rather than tapered like the RPD wax pattern, to ensure sufficient testing and information gained on the behavior of clasp material under fatigue testing. The dimension of the specimen was based on the preformed bend ring clasp wax pattern from Yeti Dental (Ring clasps bent, Art.‐Nr. 114‐0000, Yeti Dental).

**Table 1:** Materials details

Material	Manufacturer
Cast CoCr alloy	Sheralit‐cylindra CoCr, Shera
Laser‐sintered CoCr alloy	Sint‐Tech ST2724G CoCr; Parc Européen d'Entreprises
Milled PEEK	CERAMILL PEEK by Juvora™ Amann Girrbach
3D printed burnout resin	SuperCast V3, Asiga

Abbreviations: CoCr, cobalt‐chromium; PEEK, polyetheretherketone.

A sample size calculation was performed by referring to previous experiments of similar nature and outcomes using the software G*power v3.0.10 (Heinrich‐Heine‐Universitat Düsseldorf). The calculation showed that 10 specimens per group will be sufficient to show *α* = .05 and a power of 0.95 (1‐β err prob), assuming a normal distribution. To produce the cast CoCr alloy specimens, 30 burn‐out resin patterns were printed using a 3D printer (Asiga Max 3D printer) at 0° with supports. Both ends of the patterns were dipped and coated with wax to allow 3.5 mm wax‐sprues attachment and expansion before being invested with phosphate‐bonded investment (SHERACAST) using 100% expansion liquid (SHERALIQUID). The specimens were cast using a centrifugal induction casting machine (Fornex T). After casting, specimens were sandblasted with 110‐µm alumina oxide, using 2 bar air pressure (FastBlast). Thirty laser‐sintered CoCr alloy specimens were printed using a direct metal printing machine (3D Systems ProX direct metal printers, Additive Manufacturing Solution, NZ) with a build angle of 45° (Aarts et al., 2021). The PEEK specimens (*n* = 30) were milled in a milling machine (Sirona inLab MC X5) where the milling burs were changed after each puck to maintain the accuracy. The 30 specimens from each material group were randomly divided into three groups (*n* = 10 per group) based on the different deflection values of 0.25, 0.50, and 0.75 mm (the most typical undercut depth).

### 2.2 Stress at set deflection

**Figure 2:**
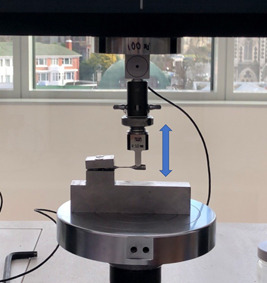
The cantilever test to establish the stress values for each specimen. The Blue arrow indicates the direction of the universal testing machine movement.

The stress (MPa) for each of the specimens at the set deflections based on the typical undercut depth of 0.25, 0.50, and 0.75 mm was determined based on the load divided by the cross‐sectional area of the gauge length of each specimen. The dumbbell end of each specimen was clamped to a cantilever jig and the opposite end was subjected to compressive load until the set deflection was reached in a universal testing machine (Instron 3369). Six load values were collected starting with the flat surface of the half‐round profile facing upwards at 0°, then rotated by 90° and finally by 180°. This was repeated by rotating the dumbbell and clamping the previously loaded end so both ends were loaded to obtain a mean stress value for each specimen before mounting in the fatigue machine, as shown in Figure 2.

### 2.3 Data analysis

Each specimen was fixed into a rotational fatigue testing machine (modified mini lathe to follow ISO:1143) with one end attached to the chuck on the motor drive unit and the other into a freely rotating chuck attached to a stage, which allowed the *x*‐ and *y*‐axis to be adjusted. An automatic counter was attached to the freely rotating end to count the cycles. When the specimen broke, the counter stopped and recorded the cycles to failure. The stage was adjusted to one of the three different deflections, 0.25, 0.50, and 0.75 mm for the specific test. The rotational speed was set to 540 rpm and the test was terminated when the specimen fractured or when 30,000 cycles, equivalent to 21 years, (Zheng et al., 2021) were completed. The stress at the specific deflection offset and the cycles to failure were used to plot stress to the number of cycles (SN graph) graph for the three materials. Following the fatigue tests, the surfaces and fracture surfaces of one specimen from each group were observed using SEM. The data obtained from the fatigue tests were statistically analyzed using the independent‐samples Kruskal–Wallis test. The statistical significance was set at *p* < .05.

## 3 RESULTS

### 3.1 Stress at set deflection

The mean stress values, before testing, for the three materials at the 0.25, 0.50, and 0.75 mm deflection are shown in Table 2. The cast and laser‐sintered CoCr specimens had statistically significantly higher stress at the three set deflections compared to the PEEK specimens (*p* < .01).

**Table 2:** The mean load, stress, and loading cycles at the set deflections for the different groups (N: Newton; S.D: standard deviation; Na: not applicable)

Specimen group (undercut depth mm)	Mean load (N)	SD	Mean stress (MPa)	SD	Mean loading cycles	SD
Cast 0.25	5.72	0.45	2.91	0.24	27,155	6277
Cast 0.50	9.37	0.97	4.77	0.33	9298	4033
Cast 0.75	14.47	1.52	7.3714	0.01	5642	8785
LS 0.25	7.10	0.64	3.62	0.52	26,765	6150
LS 0.5	13.22	1.19	6.73	0.61	11,318	7931
LS 0.75	19.32	1.92	9.84	0.02	2861	4803
PEEK 0.25	0.23	0.02	0.12	0.82	30,000	Na
PEEK 0.50	0.37	0.04	0.19	0.98	30,000	Na
PEEK 0.75	0.49	0.04	0.25	0.20	26,508	7679

Abbreviations: N, Newton; Na: not applicable; PEEK, polyetheretherketone; SD, standard deviation.

### 3.2 Fatigue strength evaluation

**Figure 3:**
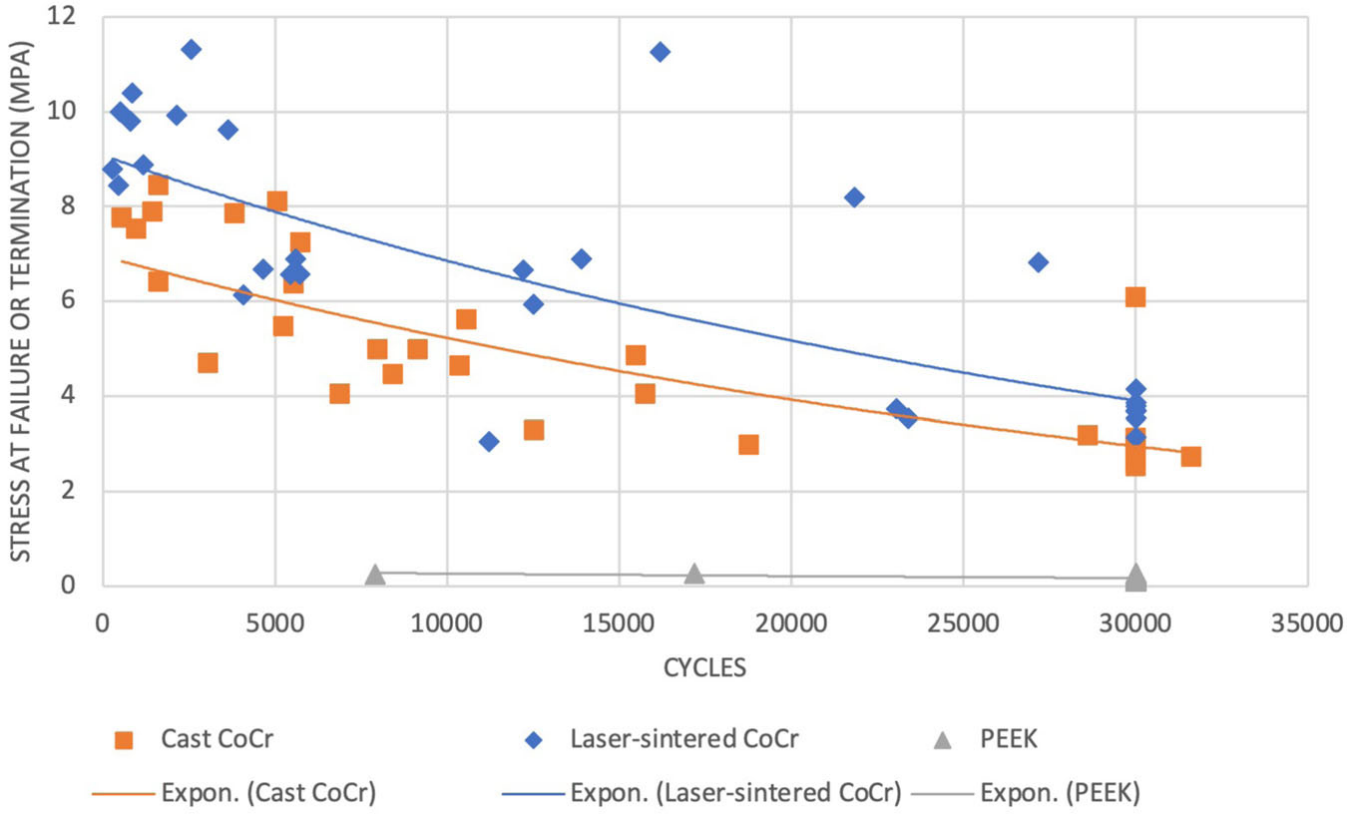
An SN curve graph for the three material groups, cast CoCr, laser‐sintered CoCr, and milled PEEK

The average numbers of cycles to failure and stress values for deflection are summarized in Table 2. Figure 3 shows the stress versus cycles (SN) curve and this graph shows a negative correlation between stress and fatigue cycles for cast CoCr and laser‐sintered CoCr specimens, which indicated that the higher the stress on the clasps, the less fatigue resistance it will have. In contrast, the PEEK specimens showed that an increase in the amount of deflection had no effect on the cycles to failure. Therefore, both the null hypotheses are rejected. Within the cast CoCr groups, 40% of the 0.25 mm deflection specimens fractured, all of the 0.5 mm deflection specimens fractured, and 90% of the 0.75 mm deflection specimens fractured. Within the laser‐sintered CoCr groups, 30% of the 0.25 mm deflection specimens fractured and all of the 0.5 and 0.75 mm deflection specimens fractured. In contrast, within the milled PEEK groups none of the 0.25 and 0.50 mm deflection specimens fractured and only 20% of the 0.75 mm specimens fractured (Table 3).

**Table 3:** The fatigue cycles of each specimen at the set deflection (NF: no failure at 30000 cycles)

Deflection (mm)	No.	Cast CoCr	Laser‐sintered CoCr	Milled PEEK
0.25	1	NF	23,040	NF
	2	NF	NF	NF
	3	12,536	23,392	NF
	4	18,777	11,213	NF
	5	21,630	NF	NF
	6	NF	NF	NF
	7	NF	NF	NF
	8	NF	NF	NF
	9	NF	NF	NF
	10	28,611	NF	NF
0.5	1	15,756	13,901	NF
	2	7984	5610	NF
	3	3056	4092	NF
	4	6901	5733	NF
	5	15,502	27,182	NF
	6	10,563	12,500	NF
	7	9151	4651	NF
	8	8443	5455	NF
	9	10,363	21,845	NF
	10	5262	12,212	NF
0.75	1	1453	281	NF
	2	5090	456	NF
	3	968	1181	NF
	4	543	2143	NF
	5	1620	3646	NF
	6	5738	16,178	NF
	7	3841	846	NF
	8	1619	808	7902
	9	5547	2565	17,182
	10	NF	510	NF

Abbreviations: CoCr, cobalt‐chromium; PEEK, polyetheretherketone.

**Figure 4:**
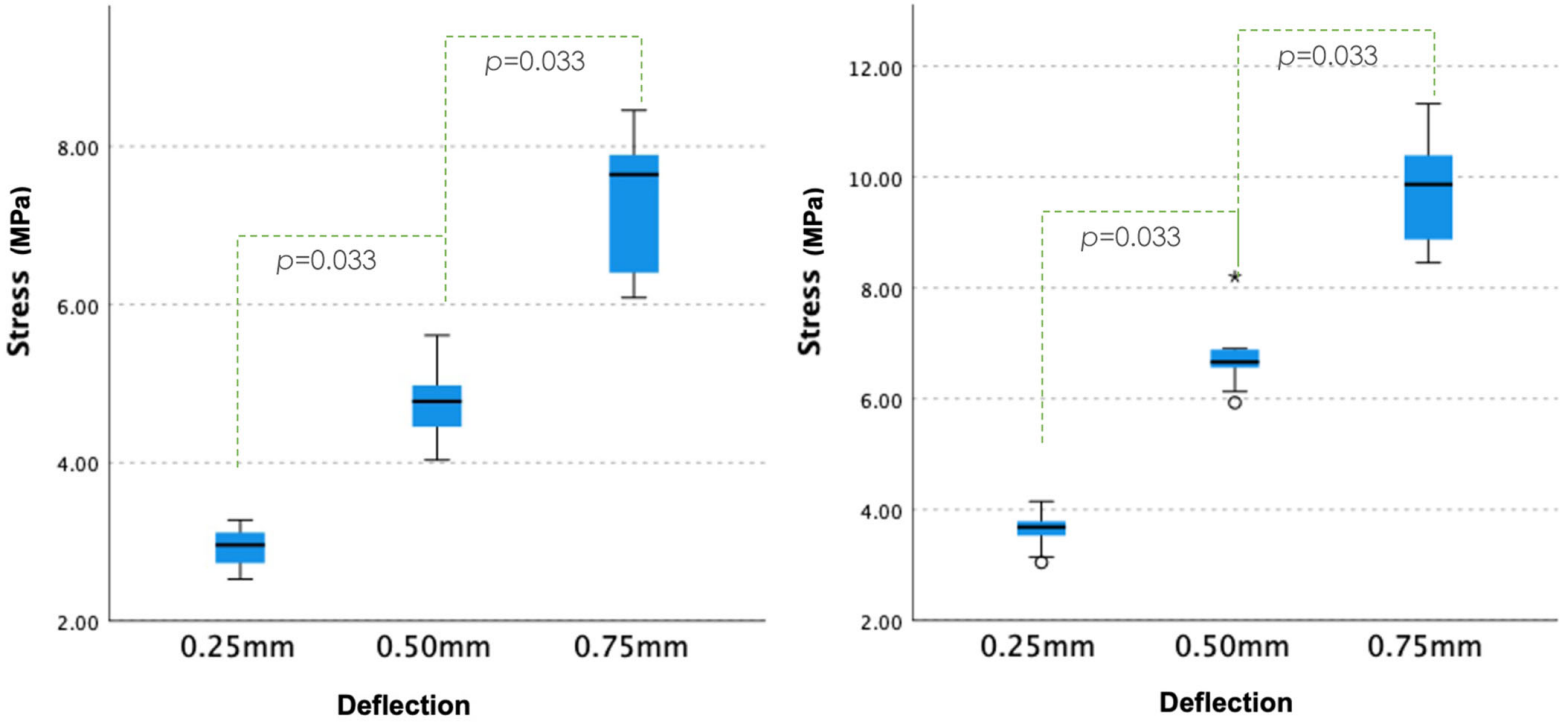
Comparison between the number of fatigue cycles and stress (N) at the failure of different materials

**Figure 5:**
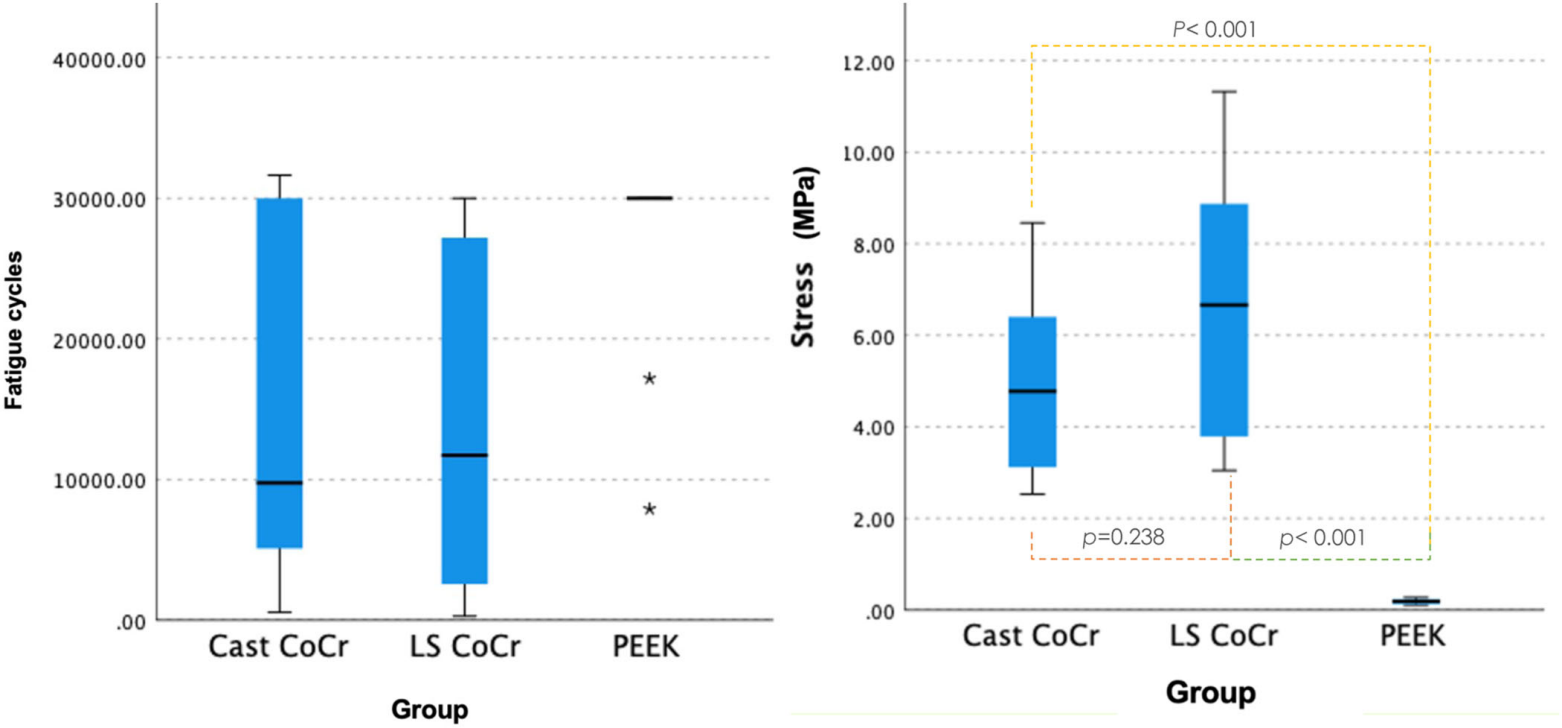
Comparisons between deflections and stress for cast and laser‐sintered cobalt‐chromium specimens

The comparison between stress and different material groups is shown in Figure 4. No statistically significant difference was observed between the cast and laser‐sintered CoCr specimens (*p* = .238). There was a statistical difference between the milled PEEK specimens and the cast and laser‐sintered CoCr specimens (*p* < .01). The comparisons between deflections and stress are shown in Figure 5. As only 6% of the PEEK specimen fractured in this study, they were excluded from the analysis. On the other hand, the cast CoCr and laser‐sintered CoCr behaved similarly. Statistically significant differences were observed across three different deflections (*p* = .033).

### 3.3 SEM analysis

**Figure 6:**
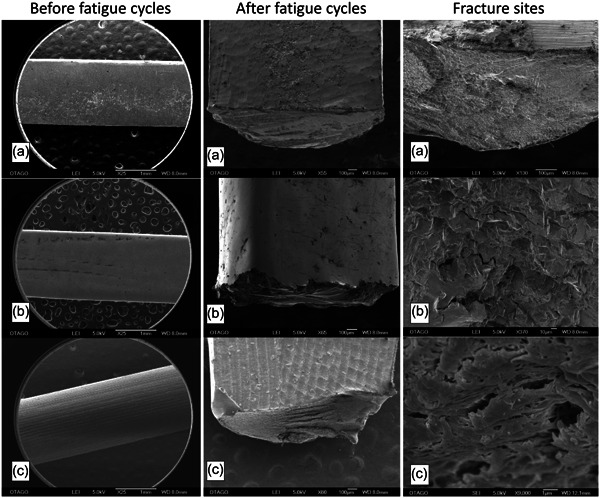
SEM images of specimen surface before and after fatigue testing and fracture sites. (a) cast cobalt‐chromium (CoCr); (b) laser‐sintered CoCr; (c) polyetheretherketone

Figure 6 shows the surface of the specimens before subjecting them to fatigue testing. Irregular casting and polishing defects were observed in the cast CoCr specimens, regular laser‐sintered defects were observed in the laser‐sintered CoCr specimens, and regular bur marks were observed in the PEEK specimens. An increase in surface defects was observed in all three materials after being subjected to fatigue testing (Figure 6). Micro‐defects radiated from the defect center and joined with other defects to propagate in the cast and laser‐sintered CoCr specimens. The surface of the PEEK specimens appeared differently compared to cast and laser‐sintered CoCr specimens. Heightened bur mark structures were observed in the PEEK specimens (Figure 6).

Figure 6 illustrates the fracture sites of cast CoCr, laser‐sintered CoCr, and PEEK specimens with high magnifications. Numerous metal dendritic structures and fatigue striation were observed in cast CoCr specimens, whereas uniform noncrystalline structures and smooth surfaces were observed in laser‐sintered specimens. In contrast, broken links of polymer chains were found in the PEEK specimens.

## 4 DISCUSSION

This study investigated the fatigue behavior of cast CoCr, laser‐sintered CoCr, and milled PEEK for RPD clasps using a novel fatigue machine. There are a number of limitations that need to be considered. First, this study only selected one type of RPD clasp dimension, as it was not viable to include all other various clasp dimensions due to cost. Second, the thickness of the specimen was derived from the average thickness of the mid‐section of a typical Akers clasp (tapered); however, the fracture location of the clasps in the clinical setting may not be at the average point. Third, this study investigated limited materials and manufacturing methods, and therefore, future studies should expand the material selections. The statistical analysis indicated that there was an association between different clasp materials and fatigue resistance, and association between undercut depth and fatigue resistance of the different clasp materials. Therefore, the first null hypothesis was rejected.

The PEEK specimens exhibited lower load at deflections than cast and laser‐sintered CoCr specimens in terms of load at deflection and retentive force. These results differ from El‐Baz et al. (2020), which stated no significant difference between PEEK and CoCr materials at 0.50 mm deflection. However, they are generally consistent with an earlier study by Tannous et al. (2012), which suggested that the PEEK material had lower retentive force than CoCr specimens. The current study did not find any statistical significance between cast and laser‐sintered CoCr specimens (*p* = .238). Our finding was different from previously published studies by Kajima et al. (2016) and Schweiger et al. (2020) they suggested the laser‐sintered CoCr is superior to cast CoCr. A possible explanation for this could be due to the different testing methodology used. The specimens in their studies were tapered instead of uniformed thickness. The location of the load and the fracture site would be different, and the tapered specimens could result in an inaccurate analysis and prediction (Murakami et al., 2021). One major drawback of Schweiger et al. (2020) study was that the study used a vertical insertion and removal method to test the retention of the clasp specimens. This study failed to mention the wear between the clasp and the tooth‐like sample as any unacceptable abrasiveness of clasps would cause irreversible damage to abutment teeth as well as failing to consider the friction forces generated during testing. Previous research indicated that the change in the coefficient of friction has a dramatic negative effect on the clasp retentive properties (Cheng et al., 2010).

In terms of the degree of deflection, cast and laser‐sintered CoCr, statistically significant differences (*p* = .033) were found across all of the three different deflections (0.25, 0.50, and 0.75 mm). These findings were consistent with Mahmoud et al. (2005) study, which suggested the probabilities of fatigue fracture and permanent deformation being closely related to the material strengths and the preset deflections. This finding was also supported by the SN curve in Figure 3, which illustrated the overall negative correlation; the higher the stress on the clasps materials, the lower the fatigue resistance. Therefore, dental professionals should endeavor to match the undercut depth and RPD clasp materials to prolong the longevity of the RPD clasping unit.

In terms of fatigue resistance, the longer the fatigue cycles, the higher the fatigue resistance the material demonstrates. The milled PEEK specimens in the current study demonstrated the highest fatigue resistance in comparison to cast and laser‐sintered CoCr specimens. Ninety‐four percent of the milled PEEK specimens did not experience failure within the 30,000‐simulation period. This result has not been mentioned in any previously published studies. While this result independently might appear positive, it should be taken in context with the other results that highlight that milled PEEK does not have sufficient retentive force when compared to cast and laser‐sintered CoCr. This is a clinically significant issue as patients may struggle to function satisfactorily with unretentive removable partial dentures. Different dimensions for the PEEK material should be investigated further in the future to understand the effect that a thicker dimension might have on milled PEEK retentive force. It would also be interesting to assess the patient‐centered outcomes in terms of their perception of comfort and aesthetic results. No statistical significance was found between cast and laser‐sintered CoCr specimens in this study (*p* = .238), which can be explained by similar material compositions. It can, therefore, be suggested that for CoCr alloy in terms of fatigue resistance behaves the same regardless of the manufacturing methods, which gives reassurance to dental professionals when selecting between cast and laser‐sintered CoCr.

In the current study, defects were observed in all specimens under the SEM. The limitation for cast CoCr specimens was that when polished manually, human errors were unavoidable. Laser‐sintered CoCr specimens had sintering defects throughout the specimens, which may have increased the percentage of having critical flaws. According to Griffith's criterion: “when brittle material is subjected to an increasing axial stress, at a particular critical stress the crack will grow spontaneously and lead to the failure of the test piece. This indicates that the larger the crack, the smaller the stress that will cause it to grow spontaneously” (Darvell, 2018). The size of the critical flaw is inversely related to the intensity of the stress necessary to initiate crack propagation. The tension and compression forces are excreted on different sides of the specimens as it rotates with deflection, which causes the crack to propagate in multiple directions. These may be the possible explanations for the low fatigue resistance and the multidirectional crack propagation pattern of the cast and laser‐sintered CoCr specimens.

The fracture site of the cast and laser‐sintered CoCr specimens had evidence of fatigue failure and brittle failure, which was represented by the fatigue striations and the catastrophic failure patterns. Fatigue failure starts with the crack tip, and stress transitions from large to smaller areas (Ơ = F/A). Higher stress causes the crack to propagate toward the least resistant pathway. The CoCr alloy has lower ductility compared to other metals (Shi et al., 1994), especially after the CoCr specimens had to undergo dislocation by the deformation of crystals. As dislocation accumulates, there is less capacity to introduce new dislocation of the crystals and plastic deformation. When no more yield is possible, brittle failure will occur and this process is referred to as work‐hardening. Additionally, work‐hardening tends to convert ductile materials into brittle materials, which may explain the failure mode of the cast and laser‐sintered CoCr specimens (Darvell, 2018). In the current study, a rapid fracture was evident at approximately two‐thirds of the cross‐sectional specimen, above the fatigue striation. A grained fracture from the fatigue crack that resulted from the dendrite was also evident. This finding is in agreement with Tokue et al. (2013) who suggested this phenomenon may be produced during the casting procedure. Broken cross‐links fibers were also found on the fracture surface of the PEEK specimens.

In terms of the microstructures at the failure site for the cast and laser‐sintered CoCr, there were notable differences. Metal dendritic structures were observed in cast CoCr specimens. The dendritic structure is formed during the slow metal solidification and weakens the structure of the specimens (Lucchetti et al., 2015). Uniformed noncrystalline structures, also known as amorphous structures and smooth metal structures, were noticed in laser‐sintered CoCr specimens. The amorphous structures are produced by rapid quenching and solidification of molten metals to bypass crystallization. Compared to traditional crystalline metals, amorphous metals have superior properties, such as high tensile strength, hardness, and wear and corrosion resistance, as suggested by Shen et al. (2017). The observation of smooth metal structure has not been previously described as it is difficult to explain this phenomenon, which might be related to the metallic abrasion before the specimen failure. Even though no statistical significance was found between the performance of the cast and laser‐sintered CoCr, the exponential trend lines in Figure 3 still demonstrated a difference. The observation of the broken crosslink polymer structure in the PEEK indicated that the material experienced high tension before catastrophic failure.

It is suggested that future studies should include fatigue behavior of RPD with different RPD clasp types, thickness, materials, as well as the optimum dimension for PEEK materials. For example, as the central region of our specimens was uniform in shape, rather than tapered like the RPD clasp (wax patterns), a future study looking into different types of specimen geometry can give better clinical relevance. It will also be beneficial to include clinical data on the longevity of RPDs made of various materials including patient outcomes.

## 5 CONCLUSION

Within the limitation of this study, it was found that cast and laser‐sintered CoCr, in terms of fatigue resistance and behaviors, showed no significant difference regardless of the manufacturing method. The results showed that all RPD materials (both cast and laser‐sintered) had sufficient fatigue resistance for clinical use in the various undercut depths. The PEEK material exhibited the highest fatigue resistance, but the low load at the set undercuts indicated that it was not a viable clasp material at this dimension to provide adequate retention.

## AUTHOR CONTRIBUTIONS

*Data collection, data interpretation, literature search, statistical analyses, and manuscript preparation*: Jenny Zheng, John M. Aarts, Sunyoung Ma, John N. Waddell, and Joanne J. E. Choi. *Study design, final approval of the manuscript*: Jenny Zheng, John M. Aarts, and Joanne J. E. Choi.

## CONFLICT OF INTEREST

The authors declare no conflict of interest.

## Data Availability

The data that support the findings of this study are available from the corresponding author upon reasonable request.
